# Safe Motherhood Case Studies: Learning with Stakeholders in South Asia—An Introduction

**DOI:** 10.3329/jhpn.v27i2.3323

**Published:** 2009-04

**Authors:** Marge Koblinsky, Nazo Kureshy

**Affiliations:** ^1^ John Snow Inc., 1616 Ft Myer Drive, Arlington, Virginia 22205, USA; ^2^ United States Agency for International Development, Washington, DC, USA

The international community resolved in 1987 to reduce maternal mortality around the world. This resolution was strengthened in 2001 when 189 countries signed the Millennium Declaration, committing themselves to Millennium Development Goal (MDG) 5 towards improvement of maternal health. To accelerate national progress towards achievement of MDG 5, a deeper understanding of what works at scale is needed. This demands a common framework for measuring progress within and across countries and learning processes that engage national stakeholders in using local evidence for programmatic decision-making, identifying critical bottlenecks in scaling up, and generating context-specific implementation solutions (1,2). However, progress has been slow and uneven. Now, as two decades ago, more than 500,000 women die each year from pregnancy-related complications—nearly half in South Asia.

“Getting on with what works”—the Lancet subtitle of an article on strategies for reduction of maternal mortality—states that we know what works to reduce the number of maternal deaths (3). The recommended priority strategy is quality intrapartum care where women deliver in health facilities staffed with a team of midwives available 24 hours a day, with a medical team at a referral hospital for back-up support in the case of life-threatening complications. This strategy has the potential to impact not only to reduce the number of maternal deaths but also mortality of newborns (4). Achieving equitable access may require innovative financing mechanisms to increase participation of care providers and women's access (5).

With leadership of the World Health Organization (WHO), the importance of such intrapartum care is now acknowledged worldwide. Countries have responded positively but implementation has varied. Many countries focus only on a part of the intrapartum care strategy: in Bangladesh, intrapartum care presently focuses on the midwifery component; in India, it is the facility component. Country and subcountry-level efforts are hampered due to lack of data to guide implementation, with even less data available to tailor strategy selection to the local context and assure active coverage and good quality (2,6). Through the case studies in this issue of the Journal, we have initiated a response to the growing call for evidence to support improved local implementation, gathering lessons from practice within and across more and less successful areas of South Asian countries. The aim is to build a body of knowledge by looking at patterns of problems and solutions to improve safe motherhood implementation at the national and subcountry levels.

## Seeking lessons for implementation of safe motherhood programmes

“Getting what works to happen” means using internationally-recognized effective strategies as a starting point rather than an end-point (6). Programmes may use knowledge of successful intervention projects and trials in their planning but rolling out depends on the interaction of policies and plans with existing formal and informal structures, procedures and practices of stakeholders, programme managers, and care providers (6). This dynamic interplay between the ideal and the existing health system is particularly important for maternal health services, which do not deliver interventions through a separate vertical programme or at one level of the health system; they rather build on all levels of the existing health system and require their interaction.

What works for safe motherhood depends not only on the service context but also on the context of recipients—their physical, social and cultural characteristics. At the individual level, maternal health encompasses many conditions—from well-being to mortality. This continuum may be traversed rapidly and without warning; complications may present as a complex (e.g. both haemorrhage and sepsis), be aggravated by underlying conditions (e.g. malaria, anaemia), and ultimately they affect at least two lives, not just one. At the family level, the response to pregnancy and birthing is mediated through a lens of joy or fear that greets having a child, the recognition and meaning given to a complication, and long-standing authority patterns that determine decisions about care-seeking. Education, poverty, traditions, and the environment (e.g. urban or rural; desert or watery; high or low density of population) are keys to understanding the community-level contextual fabric that cushions a safe motherhood programme.

A significant proportion of the variability in maternal mortality levels across and within countries may be explained by these complex interactions between care and context. Operational elements that may facilitate reduction in the number of maternal deaths have been noted in histories of successful maternal care programmes in now developed countries, such as Sweden, the United States, the United Kingdom, and Wales, and in the more recent successes in some Asian countries, such as rural China, Malaysia, Sri Lanka, and Thailand (Box 1) (8-11). Major challenges are typically the lack of available skilled care at birth and referral support, poor quality of care at birth, and lack of use of such care due to costs, distance, and other traditional barriers (5,12). Transitioning to use of skilled and referral care and the lowering of the maternal mortality ratio (MMR) can take years: Halving the MMR in developed countries, for example, typically took a decade during the mid-20th century (13).

Box 1. **Programmatic elements of successful safe motherhood programmes (9-11)**

• High availability of birthing facilities, skilled birth attendants, and relevant specialists
• Reduction of universal barriers to use (transport, costs, and perceived quality of care)
• Committed and supportive government policy that establishes the foundations for effective maternal health services (e.g. professionalization of midwifery; availability of tools—skills, supplies, time, and support; accountability; standards of care; drugs; and equipment)
• Coordination of care, linking the community, primary facilities, and hospital care, specifically emergency obstetric care, in a referral network
• Targeting of areas with disadvantaged populations with greater programme resources
• Use of information on magnitude, vulnerability, and inequities to inform policies and programmes (e.g. gathered from monitoring systems, verbal autopsies/audits, confidential inquiries, and transmitted via meetings of stakeholders and media)

South Asian countries were selected for the study as they have common elements across their health service infrastructure initiated during colonial days. And while South Asian countries currently experience relatively high levels of maternal mortality (MMR), there are also stunning pockets of success. Sites successful in reducing maternal mortality or in increasing the use of skilled birth attendance or emergency obstetric care—Tamil Nadu and Gujarat in India; Matlab and Khulna in Bangladesh—and those not as successful—northeast Bangladesh (Sylhet) and Rajasthan in India—were studied during 2004-2007.

Specific topics pursued in the case studies included barriers to/facilitators for programmatic elements—human resources, including community-based skilled birth attendants and specialists, referral units, referral systems—and the pathway leading to the primary maternal killer, bleeding (e.g. recognition of postpartum haemorrhage, pollution, blood-banks). Both primary (key-informant interviews, surveys, audits of maternal deaths, stakeholder meetings) and secondary data (documents of plans, policies; survey and facility reports; management information reports; and other extant data) were analyzed.

## Stakeholders' dialogue

Research is not enough to change policies and programmes in South Asia. Learning across both successful and less-successful sites was done together with stakeholders. Stakeholders—policy-makers and programme managers from the ministries of health at the national, state and district levels in Bangladesh, India, and Pakistan—were involved to identify areas for study, explore challenges within their systems, and share lessons across borders to stimulate thinking towards improvement of policy and programme. Solutions to implementation problems are typically multi-faceted as the challenges are complex and depend on social rather than technical intervention (e.g. programme managers may not feel that s/he has the control or authority for change). The participatory process of engagement of stakeholders fostered critical reflection and learning focused on solutions to address challenges within and beyond national and subnational borders; it enabled stakeholders to dialogue about solutions with their peers and between levels of authority for policy and programme.

Across all the sites, there were 18 meetings of stakeholders—two included officials from all the three countries. In Gujarat, for example, there were four meetings of stakeholders—three at the district level with both elected members and district health officers, and one brought together national and state-level officers from other states. The outcome was mutual learning within the state, among the states, and also between national and state-level officials.

In Bangladesh, there were four meetings of stakeholders with government officials from national and study districts; representatives from the United Nations, non-governmental organizations, and relevant professional organizations also participated. While the major constraint discussed, human resources, continues to be a bottleneck, ideas for improvements stimulated by the Tamil Nadu success are now undergoing study.

The debates, including the resulting patterns of issues and solutions that became the grist of these stakeholders' meetings (Box 2), are further detailed in the summary paper, and individual papers in this issue of the Journal (14). Such debates on issues of human resources, financing, management gaps, and availability of blood are just a beginning; much more experimenting and learning on these and other implementation issues of safe motherhood are needed, along with stakeholders' dialogue to foster change and achieve scale for safe motherhood programmes based on more localized learning.

Box 2. **Selected context-specific innovations highlighted by national leadership in regional meetings of stakeholders**

Human resources

• Cheeranjivi Yojona Scheme, a public-private partnership in Gujarat
• Incentives for deployment and retention of specialized staff in rural areas (Tamil Nadu and Kerala)
• Round-the-clock 24-hour × 7-day comprehensive EmONC centres and obstetric first-aid in PHCs (3 nurses system) in rural Tamil Nadu
• Task shifting—MBBS medical officers for comprehensive EmOC (Bangladesh)

Access, quality, and accountability

• Maternal death audit systems in Tamil Nadu and Kerala
• Equity of access through the voucher scheme (*Janani Suraksha Yojana*) and its adaptations in various states in India; voucher scheme in Bangladesh
• Centralized emergency calling services through the Emergency Medical Research Institute in Gujarat; similar services in Tamil Nadu and Andhra Pradesh; and through the Edhi Foundation in Pakistan
• Blood-banks in Maharasthra and Gujarat

Political will and leadership for safe motherhood

• Enabling environment for change: role of local champions and partnerships with professional associations in Tamil Nadu, Kerala, and Gujarat
• Training for Programme Managers: India Institute of Management
• Partnerships between institutions and researchers with higher-level national stakeholders [Indian Institute of Management; International Centre for Diarrhoeal Disease Research, Bangladesh (ICDDR,B)

EmOC=Emergency obstetric care; EmONC=Emergency obstetric and newborn care; PHCs=Primary Health Centres

We are grateful to the UK Department for International Development for funding this effort and for stimulating us to think beyond the conventional borders of topic and country. We are also grateful to the stakeholders who enriched the process of dialogue and learning with their leadership, commitment, experience, and example and who steer the course for much of what happens in country. To them we dedicate this work: Dr. P. Padmanabhan, Director of Public Health and Preventive Medicine and Director of Family Welfare of Tamil Nadu, India; Dr. Amarjit Singh, Principal Secretary, Family Welfare and Commissioner Health of Gujarat, India; Dr. Ajesh Desai, Dr. Vikas Desai, and Dr. S.R. Patel, Government of Gujarat; Dr. V. Rajasekharan Nair, Chairman, Academic Committee, KFOG of Kerala, India; Dr. Saleh Mohammad Rafique, Director, Primary Health Care and Line Director, ESD, Directorate General of Health Services, Dhaka, Bangladesh; Prof. Abdul Bayes Bhuiyan, Ex-President of Obstetrical and Gynaecological Society of Bangladesh and, Focal Point, Community-based Skilled Birth Attendant Training Programme, Dhaka, Bangladesh; Dr. Md. Nazrul Islam, Deputy Programme Manager, Reproductive Health (EOC), Directorate General of Health Services, Dhaka, Bangladesh; Dr. Abdul Majid, Special Secretary (Public Health), Health Department and Dr. Qazi Mujtaba Kamal, Provincial Coordinator, National Programme for Family Planning and Primary Health Care (Lady Health Worker Programme), Health Department, Government of Sindh, Pakistan; and Head and staff of Reproductive Health Unit, ICDDR,B, Dhaka, Bangladesh and of Indian Institute of Management, Ahmedabad, for the facilities and administrative support to the project.

## ACKNOWLEDGEMENTS

The information and views presented in this article are solely those of the authors and do not necessarily represent the views or the positions of the Department for International Development (DFID), the U.K. Government, the U.S. Agency for International Development or the U.S. Government.
